# Genes targeted by the estrogen and progesterone receptors in the human endometrial cell lines HEC1A and RL95-2

**DOI:** 10.1186/1477-7827-7-150

**Published:** 2009-12-24

**Authors:** Karin Tamm, Miia Rõõm, Andres Salumets, Madis Metsis

**Affiliations:** 1 Centre for Biology of Integrated Systems, Tallinn University of Technology, Tallinn, Estonia; 2 Nova Vita Clinic, Centre for infertility treatment and medical genetics, Tallinn, Estonia; 3 Department of Obstetrics and Gynecology, University of Tartu, Tartu, Estonia; 4 Department of Biotechnology, Institute of Molecular and Cell Biology, University of Tartu, Tartu, Estonia; 5 Competence Centre on Reproductive Medicine and Biology, Tallinn, Estonia

## Abstract

**Background:**

When the steroid hormones estrogen and progesterone bind to nuclear receptors, they have transcriptional impact on target genes in the human endometrium. These transcriptional changes have a critical function in preparing the endometrium for embryo implantation.

**Methods:**

382 genes were selected, differentially expressed in the receptive endometrium, to study their responsiveness of estrogen and progesterone. The endometrial cell lines HEC1A and RL95-2 were used as experimental models for the non-receptive and receptive endometrium, respectively. Putative targets for activated steroid hormone receptors were investigated by chromatin immunoprecipitation (ChIP) using receptor-specific antibodies. Promoter occupancy of the selected genes by steroid receptors was detected in ChIP-purified DNA by quantitative PCR (qPCR). Expression analysis by reverse transcriptase (RT)-PCR was used to further investigate hormone dependent mRNA expression regulation of a subset of genes.

**Results:**

ChIP-qPCR analysis demonstrated that each steroid hormone receptor had distinct group of target genes in the endometrial cell lines. After estradiol treatment, expression of estrogen receptor target genes predominated in HEC1A cells (n = 137) compared to RL95-2 cells (n = 35). In contrast, expression of progesterone receptor target genes was higher in RL95-2 cells (n = 83) than in HEC1A cells (n = 7) after progesterone treatment. RT-PCR analysis of 20 genes demonstrated transcriptional changes after estradiol or progesterone treatment of the cell lines.

**Conclusions:**

Combined results from ChIP-qPCR and RT-PCR analysis showed different patterns of steroid hormone receptor occupancy at target genes, corresponding to activation or suppression of gene expression after hormone treatment of HEC1A and RL95-2 cell lines.

## Background

The human endometrium is a dynamic tissue that undergoes cyclic changes in preparation for endometrial receptivity and embryo implantation. Endometrial development consists of proliferative and secretory phases, and the two major regulators of this process are the ovarian steroid hormones 17β-estradiol (E2) and progesterone (P4). In the proliferative phase, estrogens stimulate the proliferation of the epithelial and stromal components of the endometrium, while in the secretory phase P4 is involved in glandular differentiation and inhibition of E2-mediated cell proliferation [1]. In the absence of implantation, declining levels of P4 and E2 signal the degeneration of the endometrial tissue, which is followed by regeneration during the next cycle.

The biological activities of E2 and P4 are mediated mainly by nuclear receptors (NRs). Binding of a steroid hormone to its cognate receptor results in a conformational change in the NR that allows the ligand-NR complex to bind with high affinity to response elements in DNA and regulate transcription of target genes. Two types of E2 receptors, ERα and ERβ, encoded by separate genes, are found in humans [2,3]. Although ERα and ERβ are present in all endometrial cell types over the entire menstrual cycle, they are expressed at higher levels during the proliferative phase and show lower activity during the secretory phase because of the suppressive effect of P4 [4].

P4 signalling is also mediated by two receptors, PRA and PRB [5], which are encoded by the same gene but transcribed from different promoters, resulting in a PRB that has an additional 164 amino acids at the N-terminus [6]. PRB is a stronger transcriptional activator in most cell types, while PRA acts often as a dominant negative repressor for PRB activity [7,8]. PRA and PRB levels are similar during the proliferative phase. In the early secretory phase, PRA is dominant, while higher PRB levels during the mid-secretory phase have been described [9]. The expression of the PR gene in endometrial glands is controlled by E2 and P4, where E2 induces PR synthesis and P4 down-regulates the expression of its own receptor [1].

Implantation of the developing embryo involves a molecular dialogue between the endometrium and blastocyst that involves a number of specific mediators including membrane receptors, components of the extra-cellular matrix, growth factors, cytokines and lipid components of the cell membranes [10]. The endometrium is receptive for embryo attachment only during a restricted period called the "implantation window" (IW). In humans, the IW is limited to days 20-24 of the menstrual cycle and is achieved through the coordinated action of P4 and E2. Thus, an imbalance of steroid hormone levels and their ratios could influence the regulation of target genes, leading to female infertility by disturbing endometrial receptivity during the IW.

Microarray technology has led to genome-wide identification of gene expression pathways involved in implantation events. Based on five transcriptome studies [11-15], we selected 382 genes with different expression levels during the IW in human endometrium. The aim of this study was to investigate whether these pre-selected genes could be directly regulated by E2 and P4 through their specific receptors. To achieve the purpose, to study hormone dependent receptivity of the endometrium, we used two human uterine epithelial cell lines as *in vitro *models.

HEC1A was used as a model of non-receptive endometrium, and RL95-2 was used as a model of receptive endometrium [16-19]. The cell lines were chosen based on earlier studies which have demonstrated that RL95-2 cells have stronger adhesiveness for human JAR choriocarcinoma multicellular spheroids compared to HEC1A cells [20-22] and are thus considered as a model of the receptive endometrium. Following hormone treatment of the cells, binding of steroid hormone receptors to the promoters of selected genes was investigated by chromatin immunoprecipitation (ChIP) with specific antibodies, and detection of the isolated genomic sequences with quantitative PCR (qPCR). E2- and P4-dependent gene regulation was confirmed for 20 genes by reverse transcriptase (RT)-PCR.

## Methods

### Endometrial cell culture

HEC1A (#HTB-112, American Type Culture Collection, ATCC, Teddington, UK) cells were grown in McCoy 5A medium (#A1324, AppliChem GmbH, Darmstadt, Germany), while RL95-2 (#CRL-1671, ATCC) cells were maintained in DMEM/F12 (AppliChem GmbH) medium with 10 mM Hepes and 5 μl/ml insulin. Both media were supplemented with 2 g/L sodium bicarbonate (AppliChem GmbH), 10% fetal bovine serum (FBS) (AppliChem GmbH) and 1% penicillin/streptomycin (AppliChem GmbH). Both studied cell lines express E2 and P4 specific nuclear receptors (ERα, ERβ, PRA, PRB) as indicated by the ATCC. For hormonal treatment, E2 (Sigma-Aldrich, Helsinki, Finland) or P4 (4-Pregnene-3,20-dione, Sigma-Aldrich) was added to the culture media to a final concentration of 10^-8^M as described before [23,24]. For cultures with hormone supplements, dextran-coated charcoal-treated FBS and media without phenol red were used for 48 h prior to experiments to adapt similar conditions and avoid possible hormone-like (estrogenic) activity of Phenol red. Hormone treatment was 45 min for ChIP experiments and 3 h, 6 h or 12 h for mRNA experiments.

### Chromatin immunoprecipitation

Chromatin was immunoprecipitated as previously described [25], using following antibodies: monoclonal mouse anti-human ERα antibody (D-12, sc-8005); polyclonal rabbit anti-human ERβ antibody (H-150, sc-8974); monoclonal mouse anti-human PRAB antibody (AB-52, sc-810) that recognizes both human PRA and PRB receptors (AB-52, sc-810); and monoclonal mouse anti-human PRB antibody (B-30, sc-811) that recognizes only the additional NH2-terminal stretch of PRB receptor (Santa Cruz Biotech, CA, USA). Preliminary experiments determined an optimal formaldehyde cross-linking time of 15 min for both cell lines. Chromatin was fragmented to an average size of 0.5 - 2 kb using a Vibra-Cell ultrasonic processor (Sonics, Newtown, CT USA). Antibody-antigen complexes were precipitated with GammaBind™ plus Sepharose™ (GE Healthcare Life Sciences, Uppsala, Sweden), eluted with hot 0.1% SDS and uncrosslinked overnight at 65°C in the presence of Protein K (Sigma-Aldrich). DNA was purified using a Gel Extraction Kit (Qiagen, Helsinki, Finland). Control experiments used preimmune total IgG (Cell Signalling Technology, Inc, Danvers, MA, USA) to estimate the non-specific binding by unspecific antibodies.

### ChIP-qPCR for detection of genomic sequences

Specific genomic regions in the ChIP DNA samples were detected by qPCR. The 382 genomic targets for PCR were selected from previously published endometrial tissue microarray data (See additional file 1) [11-15] using two criteria: (i) ≥ 2-fold up- or down-regulated mRNA expression during IW and (ii) evolutionary conservation of human and murine orthologous sequences. Primers were designed to detect and amplify a region from +1000 to -5000 bp from the transcription start site using the Primer3 program [26] detecting NR involvement in formation of the basal transcription complex. All primers were designed to have a melting temperature (Tm) of 60°C. Primer specificity was controlled using the alignment algorithms BLAT/iPCR [27] and BLAST [28] to search the whole human genome. Primers were obtained from MWG Biotech (Edsberg, Germany). PCR was performed using HotStarTaq Master Mix (Qiagen) with 0.05 ng of template DNA and 1.25 pmol of primers for each reaction, according to the manufacturer's instructions.

PCR products were detected by qPCR using 384-well plates and a SYBR green detection method with an ABI HT7900 RT-PCR machine (Applied Biosystems, Foster City, CA, USA). PCR conditions were: HotStarTaq DNA Polymerase activation step for 14.5 min at 95°C, 40 cycles of denaturation for 30 s at 95°C, annealing for 45 s at 58°C and extension for 45 s at 72°C. A dissociation step was added to confirm the purity of PCR products by melting-curve analysis with 5 min ramping from 60°C to 95°C.

### RT-PCR

RNA was extracted using an RNeasy Mini Kit (Qiagen). Cells were washed once with phosphate-buffered saline prior to extraction, lysed directly in the tissue culture dish and homogenized by 10 strokes with an insulin syringe. RNA was prepared according to the manufacturer's protocol. Genes (n = 20, *ADAMDEC1*, *CD86*, *ETV1*, *FLT1*, *FOXA2*, *GRIP1*, *HES1*, *HOXA1*, *IHH*, *KLK3*, *MEF2D*, *MMP7*, *NCOA1*, *OTOF*, *PLXNA2*, *RELB*, *SMARCA2*, *TBX19*, *TNC*, and *ZNF54*) were chosen for RT-PCR analysis based on positive ChIP experiment results, focusing mostly on transcription factors (See additional file 2). For mRNA analysis, 5 μg of total RNA was subjected to reverse transcription, using SuperScript^® ^III First-Strand Synthesis SuperMix (#18080-400, Invitrogen Life Technologies, Carlsbad, CA, USA), as described by the manufacturer. *GAPDH *specific primers were used to normalize the cDNA synthesis with forward and reverse primers CTCTCTGCTCCTCCTGTTCGAC and TGAGCGATGTGGCTCGGCT, respectively. cDNA specific primer pairs were designed using Primer3 software and tested for unspecific priming against human genomic and mRNA sequences using iPCR software (MWG Biotech). Primers were designed to generate amplicons of 75-150 bp with a Tm of 60°C. PCR conditions for reverse transcriptase products were as described above with the exception of a 15 s annealing step at 58°C, and a 30 s extension time to account for the shorter PCR products.

### RT-PCR data analysis

Quantitative qPCR results were analysed using the publicly available software Miner [29]. Outliers were excluded manually when the mean coefficient of variation (CV) for CT (cycle threshold) for triplicates was >1%. Expression values for all transcripts were normalized to the endogenous control of *GAPDH*, and ratios relative to non-treated samples were generated. Relative gene expression ratios were calculated according to GED (Gene Expression Difference) by CT protocol [30], using the average efficiency of each gene and the CT of each triplicate. To compare the effects of hormone treatments on HEC1A and RL95-2 cell lines, statistical analysis was performed on log2-transformed values of gene expression ratios using an unpaired Student's t-test at a 95% confidence level.

### Annotation of genes

Gene classifications were carried out using the PANTHER (Protein Analysis THrough Evolutionary Relationships), [31] classification system that specifies genes by functions based on published evidence and evolutionary relationships that predict gene function in the absence of direct experimental evidence. Clustering analysis was performed with the Cluster 3.0 program [32], using uncentered correlation and complete centroid linkage. Inverted CT (CTinv) values were used for clustering qPCR data. The baseline was set to 40 and CTinv was calculated as CTinv = 40-Ct. Visualisation of clustering data was done using Java TreeView software.

Gene annotation, sequence information, gene descriptions and accession numbers (IDs) were downloaded from BioMart [33] NCBI [34] and UCSC genome browser [35] databases. All datasets were imported and kept in a database supported by MySQL database management software [36].

## Results

### E2- and P4-receptor target genes in HEC1A and RL95-2 cells

**Figure 1:**
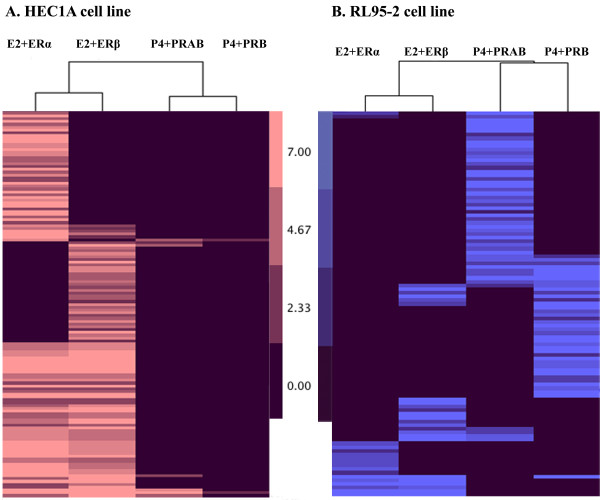
**Cluster analysis of ERα, ERβ, PRAB, PRB target genes in HEC1A and RL95-2 cells after E2 or P4 treatment**. Target genes revealed from ChIP-qPCR experiment were clustered with Cluster 3.0 program and visualized using Java TreeView software. A. HEC1A cell line. E2+ERα: 101 ERα target genes after E2 treatment; E2+ERβ: 96 ERβ target genes after E2 treatment; P4+PRAB: 7 PRAB target genes after P4 treatment; and P4+PRB: 2 PRB target genes after P4 treatment. B. RL95-2 cell line. E2+ERα: 17 ERα target genes after E2 treatment; E2+ERβ: 24. ERβ target genes after E2 treatment; P4+PRAB: 52 PRAB target genes after P4 treatment; and P4+PRB: 40 PRB target genes after P4 treatment.

Two endometrial cell lines, HEC1A and RL95-2, were used to investigate the steroid hormone-dependent binding of NRs to the promoters of 382 IW-specific genes (See additional file 1). Although the native receptive endometrium is under the combined influence of both E2 and P4, we performed hormonal treatments separately to reveal E2 or P4 dependent action through their specific nuclear receptors. Cells were treated with either E2 or P4 followed by ChIP using antibodies against the steroid hormone receptors: ERα, ERβ, PRB, or PRAB (which recognizes both PRA and PRB receptors). Quantitative qPCR with specific primers was used to detect NR-binding to the promoter region of the selected genes. HEC1A cells were treated with E2 to simulate the non-receptive-like endometrium and with P4 for comparing P4 effects on the two endometrial cell lines. Out of 382 investigated genes, 137 were ER targets: 101 were bound by ERα, 96 were bound by ERβ, and 60 were targets of both receptors (Figure 1A). When the targets common to both ERs (n = 56) and the targets shared by ERs and PRs (n = 7) were excluded, 40 genes were found to be exclusively regulated by ERα and 34 genes by ERβ (Table 1).

**Table 1:** List of unique target genes for ERα and ERβ in HEC1A cell line after E2 treatment

Gene symbol*	Gene name		
Unique targets for ERα		Unique targets for ERβ	
*ABCB10*	ATP-binding cassette, subfamily B, member 10	*AQP3*	Aquaporin 3
*ADCYAP1*	Adenylate cyclase-activating polypeptide 1	*B3GAT3*	Beta-1,3-glucuronyltransferase 3
*AGL*	Amylo-1,6-glucosidase, 4-alpha- glucanotransferase	*BMP10*	Bone morphogenetic protein 10
*APRT*	Adenine phosphoribosyltransferase	*CLU*	Clusterin
*ARID5B*	AT-rich interaction domain-containing protein 5B	*COG5*	Component of oligomeric golgi complex 5
*C3orf1*	Chromosome 3 open reading frame 1	*CRSP9*	Mediator complex subunit 7
*CCL11*	Chemokine, CC motif, ligand 11	*DMBT1*	Deleted in malignant brain tumors 1
*CCL7*	Chemokine, CC motif, ligand 7	*DNAH9*	Dynein, axonemal, heavy chain 9
*CCT6B*	Chaperonin-containing T-complex polypeptide 1, subunit 6B	*FECH*	Protoporphyria, erythropoietic
*CD86*	Cd86 antigen	*FEN1*	FLAP structure-specific endonuclease 1
*CEACAM3*	Carcinoembryonic antigen-related cell adhesion molecule 3	*FYB*	FYN-binding protein
*CLDN4*	Claudin 4	*GBAS*	Glioblastoma amplified sequence
*ECM1*	Extracellular matrix protein 1	*GPX3*	Glutathione peroxidase 3
*EDN3*	Endothelin 3	*GRIP1*	Glutamate receptor-interacting protein 1
*FBLN2*	Fibulin 2	*HBB*	Hemoglobin--beta locus
*FOSL1*	FOS-like antigen 1	*KIAA0427*	KIAA0427
*GRIK1*	Glutamate receptor, ionotropic, kainate 1	*LGTN*	Ligatin
*GSN*	Gelsolin	*MEF2D*	Myocyte enhancer factor 2D
*HOXA1*	Homeobox A1	*NINJ1*	Nerve injury-induced protein 1
*ITGB7*	Integrin, beta-7	*PDGFA*	Platelet-derived growth factor, alpha polypeptide
*ITPA*	Inosine triphosphatase	*PENK*	Proenkephalin
*LGALS8*	Lectin, galactoside-binding, soluble, 8	*PLXNB3*	Plexin B3
*MB*	Myoglobin	*PRODH*	Proline dehydrogenase
*NEK6*	Never in mitosis gene a-related kinase 6	*SC65*	Synaptonemal complex protein SC65
*NID2*	Nidogen 2	*SERPIND1*	Heparin cofactor II
*ODF2*	Outer dense fiber of sperm tails 2	*SLC16A4*	Solute carrier family 16 (monocarboxylic acid transporter), member 4
*POFUT1*	Protein o-fucosyltransferase 1	*TDG*	Thymine-DNA glycosylase
*PWP1*	PWP1 homolog (S. Cerevisiae)	*TFDP1*	Transcription factor DP1
*RELB*	v-rel avian reticuloendotheliosis viral oncogene homolog b	*TGM2*	Transglutaminase 2
*SEC61B*	SEC61 complex, beta subunit	TNC	Tenascin C
*SFRS16*	Splicing factor, arginine/serine- rich 16	*TNFRSF10C*	Tumor necrosis factor receptor superfamily, member 10c
*SGCB*	Sarcoglycan, beta	*TP63*	Tumor protein p63
*SPARC*	Secreted protein, acidic, cysteine-rich	*TRIP10*	Thyroid hormone receptor interactor 10
*SPATA2*	Spermatogenesis-associated protein 2	*WFDC2*	WAP four-disulfide core domain 2
*SSFA2*	Sperm-specific antigen 2		
*TFAP2C*	Transcription factor AP2-gamma		
*TRH*	Thyrotropin-releasing hormone deficiency		
*TRIM16*	Tripartite motif-containing protein 16		
*WNT5A*	Wingless-type MMTV integration site family, member 5A		
*XCL2*	Chemokine, C motif, ligand 2		

(*) Official gene symbol by HUGO Gene Nomenclature Committee (HGNC).

The amino acid sequence of the progesterone receptor PRA is contained within the sequence of PRB, so producing antibodies specific to PRA is not feasible. Therefore, PRA target genes were found by subtracting anti-PRB targets from the common pool of all PRAB-targets as PRB and PRAB overlapping target genes were considered to be PRB-specific. Seven target genes for P4-mediated action in HEC1A cells were identified, 5 for PRA and 2 for PRB (Figure 1A). All PR targets overlapped with ER targets and thus they were not considered to be unique targets for P4 action in HEC1A cells (Table 2).

**Table 2:** List of PRA and PRB target genes in HEC1A cell line after P4 treatment

Gene symbol*	Gene name
**Targets for PRA**	
*CD28*	CD28 molecule
*COL5A2*	Collagen, type V, alpha-2
*ETS2*	V-Ets avian erythroblastosis virus e26 oncogene homolog 2
*MYST4*	Histone acetyltransferase MYST4
*OTOF*	Otoferlin
**Targets for PRB**	
*TCF4*	Transcription factor 4
*ZNF167*	Zinc finger protein 167

(*) Official gene symbol by HUGO Gene Nomenclature Committee (HGNC).

The RL95-2 cell line was used as a model for the receptive endometrium with E2 and P4 treatments performed separately. ChIP-qPCR analysis revealed four distinct groups of target genes for each steroid hormone receptor (ERα, ERβ, PRA, and PRB) in RL95-2 cells (Figure 1B). After P4 treatment, anti-PR antibodies recognized chromatin from 83 out of the 382 potential target genes; anti PRB for 40 and anti-PRAB for 52, including overlapping targets for two antibodies. After removing common targets between PRs and ERs and subtracting anti-PRB targets from the common pool of all PRAB-targets, 37 genes were considered to be unique targets for PRA and 8 for PRB (Table 3). Although PRAB antibody should detect both PRA and PRB receptor subtypes, 31 genes were targeted solely by PRB and not identified by PRAB antibody. Twenty-five of them were found to be PRB specific, while six were additionally co-regulated by at least one of the ERs. These 25 unique PRB targets were also regarded as PRB-specific, thus resulting in 33 unique PRB target genes (Table 3). E2 treatment of RL95-2 cells resulted in 35 genes bound by ERs, where in total 17 loci were immunoprecipitated by ERα and 24 by ERβ antibody (Figure 1B), including overlapping targets for two antibodies. After deducted common targets, we discovered nine unique target genes for ERα, eight for ERβ (Table 4), and five common targets for both ERs (data not shown).

**Table 3:** List of unique target genes for PRA and PRB in RL95-2 cell line after P4 treatment

Gene symbol*	Gene name		
Unique targets for PRA		Unique targets for PRB	
*APRT*	Adenine phosphoribosyltransferase	ARID5B	AT rich interactive domain 5B (MRF1-like)
*BNIP1*	Bcl2/adenovirus E1B 19-kD protein-interacting protein 1	*ARL1*	ADP-ribosylation factor-like 1
*CCT6B*	Chaperonin-containing T-complex polypeptide 1, subunit 6B	*BRCA2*	Breast cancer 2
*CLDN4*	Claudin 4	*C2orf3*	Chromosome 2 open reading frame 3
*COL4A6*	Collagen, type IV, alpha-6	*CORT*	Cortistatin
*DNAJB1*	DNAJ/HSP40 homolog, subfamily b, member 1	*CROT*	Carnitine O-octanoyltransferase
*ECM1*	Extracellular matrix protein 1	*EIF3I*	Eukaryotic translation initiation factor 3, subunit I
*EXT1*	Exostosin 1	*ETV1*	ETS variant gene 1
*EXTL2*	Exostosin-like 2	*FGF12*	Fibroblast growth factor 12
*FGF9*	Fibroblast growth factor 9	*GADD45A*	Growth arrest- and DNA damage-inducible gene GADD45, alpha
*FLT1*	Fms-related tyrosine kinase 1	*GATA2*	GATA-binding protein 2
*GPLD1*	Phospholipase D1, glycosylphosphatidylinositol-specific	*GTF2F2*	General transcription factor IIf, polypeptide 2, 30-kD
*IFNAR2*	Interferon, alpha, beta, and omega, receptor 2	*HES1*	Hairy and enhancer of split 1 homolog
*ITGA10*	Integrin, alpha-10	*IHH*	Indian hedgehog
*ITGA2*	Integrin, alpha-2	*KLK3*	Kallikrein-related peptidase 3
*ITGB7*	Integrin, beta-7	*MEF2D*	Myocyte enhancer factor 2D
*KIAA0427*	KIAA0427	*NCR3*	Natural cytotoxicity triggering receptor 3
*LAMB1*	Laminin, beta-1	*NF1*	Neurofibromatosis, type I
*MAOA*	Monoamine oxidase A	*NFIX*	Nuclear factor I/X (CCAAT-binding transcription factor)
*MB*	Myoglobin	*NUP98*	Nucleoporin, 98-kD
*MYST4*	Histone acetyltransferase MYST4	*POSTN*	Periostin
*NCOA1*	Nuclear receptor coactivator 1	*PWP1*	PWP1 homolog (S. Cerevisiae)
*NCOR2*	Nuclear receptor corepressor 2	*RAD54L*	Rad54, s. Cerevisiae, homolog-like
*NUP155*	Nucleoporin, 155-kD	*RNF126*	Ring finger protein 126
*PDGFA*	Platelet-derived growth factor, alpha polypeptide	*SEMA3F*	Semaphorin 3F
*SLC29A2*	Solute carrier family 29 (nucleoside transporter), member 2	*SNTG1*	Syntrophin, gamma-1
*SOX4*	SRY-box 4	*SPATA2*	Spermatogenesis-associated protein 2
*TGFA*	Transforming growth factor, alpha	*SPDEF*	SAM pointed domain containing ets transcription factor
*TIAL1*	Tia1 cytotoxic granule-associated rna-binding protein-like 1	*STC1*	Stanniocalcin 1
*TNC*	Tenascin C	*STIM1*	Stromal interaction molecule 1
*TRIP10*	Thyroid hormone receptor interactor 10	*TDRKH*	Tudor and KH domains-containing protein
*TRMT11*	tRNA methyltransferase 11 homolog (S. Cerevisiae)	*TLX2*	T-cell leukemia, homeobox 2
*UBE3C*	Ubiquitin protein ligase E3C	*UMOD*	Uromodulin
*UBTF*	Upstream binding transcription factor (RNA polymerase I)		
VEGFA	Vascular endothelial growth factor A		
*XCL2*	Chemokine, C motif, ligand 2		
*ZNF549*	Zinc finger protein 549		

(*) Official gene symbol by HUGO Gene Nomenclature Committee (HGNC).

**Table 4:** List of unique target genes for ERα and ERβ in RL95-2 cell line after E2 treatment

Gene symbol*	Gene name		
Unique targets for ERα		Unique targets for ERβ	
*ANXA2*	Annexin A2	ETS2	v-ets avian erythroblastosis virus e26 oncogene homolog 2
*CHRNB2*	Cholinergic receptor, neuronal nicotinic, beta polypeptide 2	*FOXA2*	Forkhead box A2
*COL3A1*	Collagen, type III, alpha-1	*MMP26*	Matrix metalloproteinase 26
*GSN*	Gelsolin	*NINJ1*	Nerve injury-induced protein 1
*RPS6KB2*	Ribosomal protein S6 kinase, 70-kD, 2	*SERPIND1*	Heparin cofactor II
*SECTM1*	Secreted and transmembrane 1	*SFRS16*	Splicing factor, arginine/serine-rich 16
*SMARCA2*	SWI/SNF related, matrix associated, actin dependent regulator of chromatin, subfamily A, member 2	*TBX19*	T-box 19
*SC65*	Synaptonemal complex protein SC65	*VDAC1*	Voltage-dependent anion channel 1
*VLDLR*	Very low density lipoprotein receptor		

(*) Official gene symbol by HUGO Gene Nomenclature Committee (HGNC).

The total number of genes targeted after E2 treatment of HEC1A and RL95-2 cells is shown in Figure 1. Approximately four times as many genes were bound by ERs in HEC1A cells than in RL95-2 cells, which had 137 and 35 target genes, respectively. These results confirmed that HEC1A is a better model of the non-receptive endometrium with E2 exerting its primary role in tissue proliferation. In contrast, P4 treatment resulted in 10 times more PR targets in RL95-2 cells (n = 83) than in HEC1A cells (n = 7), supporting the view of RL95-2 cell line as a model for the receptive endometrium with P4 preparing the endometrium for embryo nidation.

### E2- and P4-mediated mRNA expression in HEC1A and RL95-2 cell lines

To elucidate the biological significance of the molecular interactions seen by ChIP, we complemented the initial results with mRNA expression studies for 20 genes (See additional file 2). All selected genes were detected as NR targets by ChIP-qPCR and half (n = 10) of them were classified as transcription factors. The other criteria after transcription factors was to find genes which has not been described as an ER or PR targets before.

HEC1A and RL95-2 cells were treated with E2 or P4 for 3, 6, or 12 h. At least 2-fold up- or down-regulation of mRNA expression levels were detected for 12 genes (*CD86*, *FOXA2*, *IHH*, *MEF2D*, *MMP7*, *NCOA1*, *OTOF*, *PLXNA2*, *RELB*, *SMARCA2*, *TNC*, and *ZNF549*) out of 20 genes studied (Table 5). Although, the activities of transcription factor coding genes *GRIP1 *and *TBX19 *changed significantly during the hormonal treatments, these alterations remained below the 2-fold threshold. cDNA amplification was not observed for 6 genes *ADAMDEC1*, *ETV1*, *FLT1*, *HES1*, *HOXA1 *and *KLK3 *in either cell line regardless of hormonal treatment (data not shown). Surprisingly, the observed changes in E2- or P4-dependent mRNA expression occasionally conflicted with the ChIP-qPCR results. For example, although the secreted growth factor Indian hedgehog (*IHH*) and otoferlin (*OTOF*) were detected as E2-ER targets in HEC1A cells, E2-induced mRNA expression was observed in RL95-2 cells but not in HEC1A cells.

**Table 5:** Genes > 2 fold up- or down-regulated after hormone treatment in HEC1A and/or RL95-2 cell line

Up-regulation > 2 fold			ChIP target post E2 treatment		ChIP target post P4 treatment
HEC1A	Molecular function*	E2		P4	
*MEF2D*	Other transcription factor; Nucleic acid binding	6 h	HEC1A ERβ	3 h	RL95-2 PRB
RL95-2		E2		P4	
*CD86*	Immunoglobulin receptor family member; Membrane-bound signaling molecule; Defense/immunity protein	3 h	HEC1A ERα	NS	ND
*MEF2D*	Other transcription factor; Nucleic acid binding	3 h	HEC1A ERβ	3 h	RL95-2 PRB
***OTOF***	Otoferlin	12 h	HEC1A ERα and ERβ	3 h	HEC1A PRA
***PLXNA2***	Tyrosine protein kinase receptor;Protein kinase	3 h	RL95-2 ERβ	3 h	RL95-2 PRA
*RELB*	Other transcription factor	3 h	HEC1A ERα	NS	ND
***SMARCA2***	DNA helicase	3 h	RL95-2 ERα	3 h	ND
*TNC*	Cell adhesion molecule; Extracellular matrix glycoprotein	3 h	HEC1A ERβ	3 h	RL95-2 PRA

**Down-regulation > 2 fold**					
HEC1A	Molecular function*	E2		P4	
*CD86*	Immunoglobulin receptor family member; Membrane-bound signaling molecule; Defense/immunity protein	6 h	HEC1A ERα	NS	ND
*FOXA2*	Other transcription factor; Nucleic acid binding	3 h	RL95-2 ERβ	12 h	ND
***MMP7***	Metalloprotease; Other extracellular matrix	12 h	HEC1A ERα and ERβ	12 h	ND
*NCOA1*	Transcription factor; Acetyltransferα se	3 h	ND	6 h	RL95-2 PRA
*RELB*	Other transcription factor	3 h	HEC1A ERα	6 h	ND
*TNC*	Cell adhesion molecule; Extracellular matrix glycoprotein	3 h	HEC1A ERβ	6 h	RL95-2 PRA
*ZNF549*	KRAB box transcription factor		HEC1A ERα and ERβ	6 h	RL95-2 PRA
RL95-2		E2		P4	
*CD86*	Immunoglobulin receptor family member; Membrane-bound signaling molecule; Defense/immunity protein	12 h	HEC1A ERα	NS	ND
***IHH***	Other signaling molecule; Protease	12 h	HEC1A ERα/ERβ	6 h	RL95-2 PRB

(*) Molecular functions were obtained from PANTHER. In bold are genes with cell line-specific expression. NS: not significant gene expression, ND: not detected. Official gene symbols according to HUGO Gene Nomenclature Committee (HGNC).

From the selected 20 genes five genes showed significant cell line-specific mRNA expression either in HEC1A or RL95-2 cell line (Table 5, in bold). *IHH *expression was detected in RL95-2 cell line-specific manner until 6 h after E2 treatment or 3 h after P4 treatment, but was down-regulated after longer hormonal exposure. In addition to *IHH*, the expression of three other genes: plexin A2 (*PLXNA2*), *OTOF *and a SWI/SNF related family member (*SMARCA2*) were induced by E2 or P4 in the RL95-2 cell line. In HEC1A cells, on the contrary, the expression of matrix metalloprotease 7 (*MMP7*) was detected in non-treated HEC1A cells and was gradually down-regulated after treatments by both hormones.

**Figure 2:**
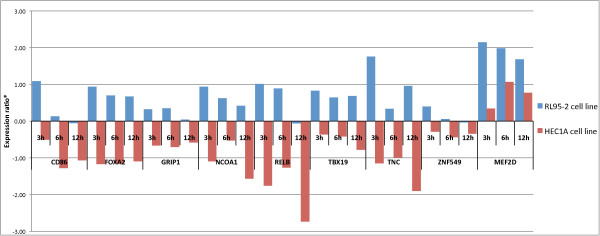
**E2 mediated time-dependant gene expression in RL95-2 (blue) and HEC1A (red) cell lines compared to non-treated samples**. Expression profiles of *CD86*, *FOXA2*, *GRIP1*, *NCOA1*, *RELB*, *TBX19*, *TNC*, *ZNF549*, and *MEF2D *genes had significant difference (p < 0.05, two-tailed, unpaired Student's t-test with 95% confidence of log2-transformed expression ratios relative to baseline expression) in mRNA expression ratios (*) between RL95-2 and HEC1A cells after E2 treatment. E2 exposure predominantly induced mRNA level of gene expression in RL95-2 cells but down-regulated in HEC1A cells (except for *MEF2D *gene).

**Figure 3:**
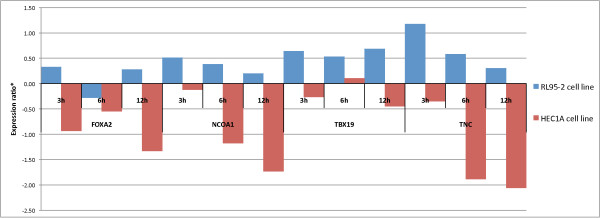
**P4 mediated time-dependant gene expression in RL95-2 (blue) and HEC1A (red) cell lines compared to non-treated samples**. Expression profiles of *FOXA2, NCOA1, TBX19 *and *TNC *genes had significant difference (p < 0.05, two-tailed, unpaired Student's t-test with 95% confidence of log2-transformed expression ratios relative to baseline expression) in mRNA expression ratios (*) between RL95-2 and HEC1A cells after P4 treatment. P4 mostly induced mRNA level in RL95-2 cells but down-regulated in HEC1A cells.

Analysis of gene expression in HEC1A and RL95-2 cell lines showed almost opposite responses to E2 and P4. A significant difference (p < 0.05) in transcript levels was observed between the two cell lines in the expression of nine genes after E2 treatment (Figure 2). Furthermore, *CD86*, *FOXA2*, *GRIP1*, *NCOA1*, *RELB*, *TBX19*, *TNC*, and *ZNF549 *genes showed opposite regulation in the two cell lines, with mRNA levels up-regulated in RL95-2 cells but down-regulated in HEC1A cells after E2 treatment when compared to non-treated samples (Figure 2). The expression of *MEF2D *was also significantly different between the cell lines, but unlike the other genes, it was up-regulated by E2 in both cell lines. P4 similarly exerted opposite effects on gene expression in the two endometrial cell lines as mRNA levels of the three transcription factor genes *FOXA2*, *NCOA1*, *TBX19*, and extracellular matrix tenascin-C (*TNC*) gene were significantly (p < 0.05, Figure 3) up-regulated in RL95-2 cells and down-regulated in HEC1A cells post P4 treatment.

## Discussion

The exact molecular characteristics of the embryo impantation are still not completely characterised because of the complexity of using human embryos and endometrial tissue in reseach, therefore other means must be elucidated for research of receptivity of the endometrium. Used RL95-2 and HEC1A cell lines are both described as endometrial epithelial cell lines derived from adenocarcinoma cells. Our interest to investigate the suitability of selected cell lines as an *in vitro *models of non-receptive and receptive endometrium was based on several studies published recently [21,22,37,38]. RL95-2 cell line has been characterized as a model of receptive endometrium by its ability to mimic relevant properties of the adhesion competent endometrial lining compared to HEC1A [21,22].

The steroid hormones E2 and P4 bind to NRs to play a key role in preparing the endometrium for implantation of an embryo. NRs regulate transcription by binding to the proximal or distal regulatory regions of specific genes. Transcriptional changes regulated by NRs have been suggested to involve chromosomal looping or PolII tracking that allows enhancers to interact with the transcription complex at the promoter [39]. In this study, we used ChIP-qPCR method to investigate whether genes previously identified as IW-specific become direct targets of NRs after hormonal treatment of endometrial cells.

Combining new technologies based on microarrays and high-throughput sequencing with the today's knowledge of entire human genome allows defining of all *in vivo *targets for transcription machinery in a single experiment [23,24]. Since NR activity is often tissue specific, we used primers specifically designed to detect the promoter regions of 382 genes known to exhibit differential expression in the human receptive endometrium. Our specific aim was to investigate whether the steroid hormone actions in endometrial cell lines HEC1A and RL95-2 support their use as non-receptive and receptive endometrial models, respectively.

In non-receptive endometrium, the primary role of E2 is in tissue regeneration and proliferation. Analysis of the number of ER target genes identified by ChIP-qPCR after E2 treatment under our experimental conditions, revealed almost 4-fold difference between HEC1A (n = 137) and RL95-2 (n = 35) cells. This finding supports the idea of HEC1A as a good model for non-receptive endometrium being predominantly governed by E2 control. In addition to unique targets of ERα and ERβ, we identified a subset of 60 genes in HEC1A cells and 5 genes in RL95-2 cells that were common for both ERs.

Previous *in vitro *studies have demonstrated that both homodimers ERα/ERα or ERβ/ERβ and heterodimers ERα/ERβ can be formed when both ER subtypes are expressed in the same cell [40]. Moreover, it has been shown that in the absence of ERα, ERβ can either inhibit ERα-mediated gene transcription or partly replace ERα as a transcription factor [41]. Thus, the common ERα and ERβ targets identified in this study could represent genes either activated by heterodimeric complexes or could reflect competitive binding of the two separate E2 receptors to the same regulatory element.

In receptive endometrium, the primary role of P4 is the preparation of the endometrium for embryo implantation. From our set of 382 genes, 83 were bound by PRs in RL95-2 cells, which was ten times more than in the HEC1A cell line (n = 7). Harduf and colleagues have demonstrated that there is a higher PRA/PRB ratio in RL95-2 cells compared to HEC1A cells indicating the possible important role of PRA during implantation window [22]. Even the both cell lines express E2 and P4 specific receptors (ATCC) the lack of specific antibody against PRA has made it difficult to compare the exact ratio levels of two PRs.

To investigate transcriptional changes that occur after NR binds to its promoter region, we assessed the expression of 20 selected genes. RT-PCR analysis showed that 12 out of selected 20 IW-specific genes had at least 2-fold up- or down-regulation in mRNA expression level after hormonal treatment. Five genes were expressed in cell line-specific manner as the expression of *IHH*, *PLXNA2*, *OTOF*, and *SMARCA2 *was only observed in RL95-2 cells, while *MMP7 *activity was detected exclusively in HEC1A cells.

We included *IHH *as a classical example of steroid hormone-dependent gene regulation during IW. Previous *in vivo *and *in vitro *studies revealed that the expression of *IHH *in the murine uterus is solely P4 dependent and essential for embryo implantation [42-44]. Our results showed that *IHH *was the target gene for PRB after P4 treatment in RL95-2 cells. Indeed, RT-PCR analysis confirmed *IHH *expression only in RL95-2 cells. Interestingly, the mRNA levels of *IHH *in RL95-2 cells were suppressed after the use of either E2 or P4 hormones (Table 5), differentially from murine uterus where *IHH *expression depends only on P4 [42-44]. Yet, the recent work has indicated that the stromal not epithelial PR is critical for P4-mediated induction of *IHH *expression in the mouse uterus [45]. Since the HEC1A and RL95-2 cell lines represent the epithelial cells of the endometrium, *IHH *expression in this endometrial compartment may be regulated by other hormonal mechanisms from internal tissue layers. Species difference between the estrous cycle of mice and the menstrual cycle in women is an additional factor to account for this discrepancy.

Our gene expression analysis focused mainly on transcription factors. Since the development and regeneration of endometrial tissue is largely governed by E2 and P4, a transcriptional regulatory feedback system is needed to mediate these dynamic and complex changes. The majority of E2-regulated genes are thought to be up-regulated after short (1-8 h) hormonal treatment, while most of the hormone-responsive genes are down-regulated after longer (12-48 h) treatment [39]. We examined the changes in mRNA expression following 3-12 h of hormonal exposure in order to investigate the rapid transcriptional changes caused by steroid hormones. We found that the increase of mRNA level in selected genes mainly occurred after short-term (3-6 h) treatment, while the majority of suppressive effects become visible after 6-12 h of treatment (Table 5, Figures 2 and 3).

Interestingly, when hormone responsiveness in two endometrial cell lines was compared, we found that hormonal treatments had almost opposite effects on gene expression. Treatments with E2 or P4 resulted in significant up- and down-regulation of genes in RL95-2 and HEC1A cells, respectively. Genes for the transcription factors *FOXA2*, *NCOA1*, *TBX19*, and the extracellular matrix glycoprotein *TNC *had markedly different and opposite mRNA expression levels after E2 or P4 treatment in both of the cell lines investigated. All four genes were either down-regulated by both hormones in HEC1A or up-regulated in RL95-2 cells (Figures 2 and 3). In addition to already mentioned genes, E2 had also an inverse effect on *CD86*, *GRIP1*, *RELB*, and *ZNF549 *mRNA expression in the two endometrial cell lines, up-regulated in RL95-2 and down-regulated in HEC1A cells.

Some of the genes (*ADAMDEC1*, *ETV1*, *FLT1*, *HES1*, *HOXA1 *and *KLK3*) did not show any evidence of gene activity in both cell lines in spite of hormonal treatment. As all genes were selected from previous expression studies with human endometrium tissue samples the revealed effect could be cell line specific. In addition, we have to take into account that NR binding alone is not always sufficient to induce gene expression changes. The various combinations of transcription co-activators or -repressors are also required for proper gene activity, depending on the specific cell and tissue background [46-48]. In studying steroid hormone signalling, the non-genomic actions of steroid hormones in cross-talk between the growth factor receptors and cytoplasmic response must also be considered along with the direct transcriptional effects mediated by NRs [49,50].

In present study we gained new information about the specific action of E2 and P4 during different stages of human endometrial development using a combination of ChIP-qPCR and RT-PCR. HEC1A and RL95-2 cell lines showed different sets of ER and PR target genes in response to hormonal stimuli as more target genes were detected for ERs in HEC1A cells than for PRs in RL95-2 cells. We also observed that hormone treatment had different impacts on gene expression levels in the two cell lines. The ChIP and RT-PCR results show that E2 and P4 actions in the human endometrium are more complex than the classic steroid hormone effects mediated through NRs.

## Conclusions

The presented data demonstrates that the endometrial cell lines HEC1A and RL95-2 are suitable *in vitro *models for evaluating the effects of steroid hormones in non-receptive and receptive endometrium, respectively. This study deepens our understanding on the hormone responsive gene regulation during the cyclic changes in the human endometrium. However, further studies are needed to elucidate the complex mechanism by which endometrium acquires its receptivity.

## Competing interests

The authors declare that they have no competing interests.

## Authors' contributions

KT carried out experimental studies and prepared the draft version of the manuscript. MR participated in experimental work and performed data analysis of ChIP and mRNA expression studies. MM conceived the study, mainly designed and coordinated the work and performed the bioinformatic analysis. AS contributed to the design of the study and helped with the manuscript preparation. All authors read and approved the final version of the manuscript.

## Supplementary Material

Additional file 1 **Supplementary Table 1**. Genes used in ChIP-qPCR for ER and PR binding site analysis. Click here for file

Additional file 2 **Suplementary Table 2**. Genes used in mRNA analysis. Click here for file
